# The role of PML ubiquitination in human malignancies

**DOI:** 10.1186/1423-0127-19-81

**Published:** 2012-08-30

**Authors:** Ruey-Hwa Chen, Yu-Ru Lee, Wei-Chien Yuan

**Affiliations:** 1 Institute of Biological Chemistry, Academia Sinica, Taipei, Taiwan; 2 Institute of Biochemical Sciences, College of Life Science, National Taiwan University, Taipei, Taiwan

**Keywords:** PML, Ubiquitination, Tumor suppression

## Abstract

Tumor suppressors are frequently downregulated in human cancers and understanding of the mechanisms through which tumor cells restrict the expression of tumor suppressors is important for the prognosis and intervention of diseases. The promyelocytic leukemia (PML) protein plays a critical role in multiple tumor suppressive functions, such as growth inhibition, apoptosis, replicative senescence, suppression of oncogenic transformation, and inhibition of migration and angiogenesis. These tumor suppression functions are recapitulated in several mouse models. The expression of PML protein is frequently downregulated in diverse types of human tumors and this downregulation often correlates with tumor progression. Recent evidence has emerged that PML is aberrantly degraded in various types of tumors through ubiquitination-dependent mechanisms. Here, we summarize our current understanding of the PML ubiquitination/degradation pathways in human cancers. We point out that multiple pathways lead to PML ubiquitination and degradation. Furthermore, the PML ubiquitination processes are often dependent on other types of posttranslational modifications, such as phosphorylation, prolylisomerization, and sumoylation. Such feature indicates a highly regulated nature of PML ubiquitination in different cellular conditions and cell contexts, thus providing many avenues of opportunity to intervene PML ubiquitination pathways. We discuss the potential of targeting PML ubiquitination pathways for anti-cancer therapeutic strategies.

## Review

### Introduction

The *PML* gene was originally identified at the breakpoint of the *t*(15;17) chromosomal translocation that is found in most cases of acute promyelocytic leukemia (APL) [1]. This translocation generates PML-RARα fusion protein, which plays a driving role in APL pathogenesis. Since its discovery, the *PML* gene has been subject to intensive studies. The human *PML* gene can give rise to at least 12 transcripts due to alternative splicing [2,3]. These isoforms share an identical N-terminal region containing RBCC (Ring, B-box, and coiled-coil) motif but differ in their C-termini. PML is the essential component of PML-nuclear bodies (PML-NBs), which are spherical subnuclear organelles with a diameter of 0.1 to 1 μm [4,5]. PML functions as the organizing center of this structure, allowing the recruitment of numerous proteins through mechanisms involving SUMO-modifications and interactions [6]. PML-NBs are known to fine tune a number of cellular processes and most of them are related to tumor suppression [4]. As PML is crucial for the assembly of PML-NBs, the biological functions of PML-NBs are somewhat difficult to be distinguished from those of PML.

### PML tumor suppressive functions

The tumor suppressive function of PML was first suggested by the identification of PML-RARα fusion protein in APL. Additional genetic evidence comes from PML null mice. These mice are prone to develop papilloma, carcinoma, and T/B lymphomas in several chemical and physical modes of carcinogenesis [7]. When crossed with other tumor mouse models, PML loss accelerates tumor development [8]. Furthermore, studies with in vitro and/or in vivo systems have revealed a number of PML-elicited cellular functions that are related to tumor suppression. These functions are described below:

 1.*Regulation of apoptosis:* Many lines of evidence indicate a role of PML in modulating apoptosis. For instance, lymphocytes, thymocytes and embryonic fibroblasts derived from *PML*^*−/−*^ mice are more resistant to apoptosis induced by stimuli that activate either intrinsic or extrinsic apoptotic pathway, compared with their wild type counterparts [9,10]. The pro-apoptotic functions of PML can be mediated by both p53-dependent and p53-independent mechanisms. PML activates p53 by multiple mechanisms, such as promoting its phosphorylation and acetylation by recruiting it into PML-NBs and by binding and inhibiting the p53 negative regulator MDM2 [11,12]. Besides p53, several other factors are implicated in the pro-apoptotic function of PML. One such molecule is Daxx, which is recruited to PML-NBs through its interaction with sumoylated PML [13]. Daxx is reported to repress the expression of several anti-apoptotic genes when localized in PML-NBs, thereby eliciting pro-apoptotic functions [14]. Recently, the extranuclear PML has been reported to form a complex with inositol 1,4,5-triphosphate receptor (IP3R), Akt and PP2a in ER and mitochondria-associated membrane, where PML participates in Akt- and PP2a-dependent modulation of IP3R phosphorylation to promote IP3R-mediated calcium release from ER, thereby inducing apoptosis [15].

 2. *Regulation of cellular senescence:* PML was first implicated in cellular senescence by its requirement for V-H-Ras-induced senescence via modulating p53 acetylation and activation [16]. Subsequently, it has been found that overexpression of a particular PML isoform, PML-IV, induces senescence through an Rb-dependent mechanism [17,18]. Intriguingly, during the induction of senescence, PML-NBs are colocalized with senescence-associated heterochromatin foci (SAHF), Rb, and E2F, which is thought to mediate PML-induced repression of E2F target genes, leading to proliferation arrest, DNA damage and senescence [19]. Recent study identified TBX2, a T-box transcription factor, as an E2F target critical for PML-induced senescence [20].

 3. *Regulation of neoangiogenesis:* PML deficiency leads to increased neoangiogenesis and elevated expression of pro-angiogenic factors such as HIF-1α and VEGF in human and mouse tumors. PML inhibits angiogenesis by negatively regulating the Akt-mTOR pathway, which controls the protein synthesis of HIF-1α. PML can recruit PP2a to PML-NBs, thereby dephosphorylating and inactivating Akt [21]. In addition, PML recruits mTOR activator Rheb to the nucleus, thereby inhibiting mTOR [22]. The ability of PML to regulate mTOR/HIF-1α pathway also implicates its function in hypoxia responses.

 4. *Regulation of cell migration:* PML was recently found to inhibit the migration of MDA-MB231 breast cancer cell lines by downregulating the expression of integrin β1. This finding also implicates a role of PML in suppressing tumor metastasis [23].

 5. *Regulation of DNA damage responses:* PML has been implicated in regulating DNA damage responses by targeting a number of effectors of these pathways to PML-NBs [24]. In response to DNA damage, the number and size of PML-NBs are increased in an ATM- and ATR-dependent manner [25]. However, the functional consequence of this phenomenon is currently unknown.

### Regulation of PML expression in human cancers

The pleiotropic functions of PML in tumor suppression suggest that inactivation or downregulation of PML would provide an advantage for tumor development and progression. Indeed, in addition to the disruption of PML function by PML-RARα fusion protein in APL, complete or partial loss of PML protein expression has been observed in human cancers from multiple origins, such as prostate adenocarcinoma, colon adenocarcinoma, breast carcinoma, lung carcinoma, lymphoma, CNS tumors, and germ cell tumors. In certain types of tumors, such as prostate cancer, PML loss correlates with invasive and metastatic progression. Interestingly, despite the frequent downregulation of PML protein in tumors, PML mRNA is expressed in all tumor samples and cell lines tested. This study further showed that treatment of several PML-negative tumor cell lines with proteasome inhibitor leads to re-expression of PML protein and restoration of PML-NBs [26]. Thus, proteasome-dependent degradation is proposed to be a mechanism by which tumor cells restrict the expression of PML. Since ubiquitination is the major posttranslational modification that controls protein degradation through proteasome, identification of PML ubiquitin ligases would provide insights into the mechanism of PML regulation in human cancers.

### RNF4-mediated PML ubiquitination

Arsenic trioxide (ATO) is the leading choice of anti-APL therapeutic agent [27]. Mechanistically, ATO triggers PML-RARα degradation through its PML moiety and sumoylation at K160 residue is necessary for targeting PML-RARα and PML to the proteasome [28]. Interestingly, ATO directly binds to several adjacently localized cysteine residues in the PML RING domain to facilitate PML oligomerization [29]. In addition, ATO-induced ROS production also contributes to PML intermolecular cross linking by disulfide bonds [30]. Oligomerization of PML facilitates its targeting to PML-NBs and enhances its interaction with SUMO conjugating enzyme Ubc9, thus facilitating PML hypersumoylation [29]. A recent study indicates an involvement of SUMO E3 ligase PIAS1 in ATO-triggered PML sumoylation [31]. The molecular mechanism governs sumoylation-induced PML ubiquitination has been elucidated. A family of SUMO targeted ubiquitin ligases (STUbLs) was discovered by their harboring of a RING domain and multiple SUMO-interacting motifs (SIMs). This structure feature allows STUbLs to specifically recognize sumoylated substrates [32]. RNF4 is a STUbL-family E3 ligase and is rapidly recruited to PML-NBs following ATO treatment through a SUMO-dependent mechanism [33]. With its multiple SIMs, RNF4 interacts strongly with polysumoylated PML, thereby facilitating PML K48 poly-ubiquitination and proteasomal degradation [34,35]. RNF4 depletion leads to accumulation of polysumoylated PML and impairment of ATO-triggered PML degradation. Among the three sumoylation residues of PML, K160 is most critical for RNF4 recruitment and ATO-induced proteolysis [35]. This SUMO-coupled ubiquitination mechanism also applies to PML-RARα. Accordingly, primary haematopoietic cells transformed by K160 mutant of PML-RARα are resistant to ATO-induced terminal differentiation. Dominant-negative RNF4 similarly blocks this ATO-induced differentiation. Thus, RNF4-mediated PML-RARα ubiquitination and degradation plays a vital role in the APL therapeutic response to ATO.

### KLHL20-mediated PML ubiquitination

KLHL20 is a member of the BTB-kelch family proteins. Similar to many members of this family, KLHL20 forms a ubiquitin E3 ligase complex with Cullin3 and Roc1 and functions as the substrate binding component of this ligase. The first linkage between KLHL20 and PML is the observation that a small fraction of KLHL20 is distributed to PML-NBs under normal growth conditions. This distribution is further augmented with the increase of PML transcription by interferon [36]. The expression of KLHL20 is induced under hypoxia conditions through a HIF-1-dependent manner and this KLHL20 induction correlates with downregulation of PML protein expression. Subsequent study indicates that the KLHL20-based E3 ligase complex mediates hypoxia-induced PML ubiquitination and proteolysis. However, targeting PML to KLHL20 requires two posttranslational modifications. The first one involves phosphorylation of PML at S518 by CDK1 and CDK2. The S518-phosphorylated PML is then recognized by peptidylprolyl *cis/trans* isomerase Pin1 and Pin1-catalyzed isomerization further potentiates PML interaction with KLHL20. Importantly, this hypoxia-induced, KLHL20-mediated PML ubiquitination pathway not only attenuates PML tumor suppressive functions but also participates in a feedback mechanism to maximize the production of HIF-1α during hypoxic stress. Consequently, KLHL20-PML pathway amplifies multiple tumor hypoxia responses, such as metabolic reprogramming, epithelial-mesenchymal transition, migration, tumor growth, angiogenesis, and chemoresistance, and these functions collectively lead to aggressive tumor phenotypes. Clinically, overexpression of HIF-1α, KLHL20, Pin1 and downregulation of PML are found in prostate cancers. While the expression of HIF-1α positively correlates with that of KLHL20, PML expression inversely correlates with the expression of HIF-1α, KLHL20 and Pin1. More importantly, the HIF-1α high, KLHL20 high, Pin1 high and PML low expression profile correlates with high-grade tumors. These clinical data strongly suggest the existence of this PML destruction pathway in prostate cancer and the association of its hyperactivation with disease progression [37]. Thus, the Roc1-Cullin3-KLHL20 complex represents the first PML ubiquitin ligase that is dysregulated in human cancers and the KLHL20-mediated PML destruction and HIF-1α feedback regulation contribute significantly to tumor progression.

### E6AP-mediated PML ubiquitination

E6AP is the founding member of the HECT-family ubiquitin ligases [38], in which ubiquitin is covalently bound to the HECT domain before transferring to substrates [39]. E6AP is partially localized in PML-NBs and is physically associated with PML. Consistent with these features, E6AP promotes ubiquitination and proteasomal degradation of PML. The physiological role of E6AP in PML degradation is highlighted by the finding that multiple organs of E6AP null mice display elevated PML protein level. The increased level of PML protein and PML-NBs are also evident from E6AP-deficient mouse embryonic fibroblasts. Furthermore, lymphoid cells derived from such mice accumulate PML protein in response to DNA damage and are more susceptible to DNA damage-induced death, implying a role of this PML degradation in cell response to genotoxic stress [40]. More direct evidence for the function of E6AP-mediated PML degradation in tumorigenesis comes from the study of Myc-induced B-cell lymphomagenesis. The *E*μ*-myc* transgenic mice represent a well established model for pre-B/B lymphoma development [41,42]. Importantly, loss of one allele of E6AP delays the onset of Myc-induced pre-B/B cell lymphoma and reduces the tumor burden. Mechanistically, the suppression of lymphomagenesis by E6AP heterozygosity is attributed to the increased senescence of pre-B/B lymphoma cells, as evident by the elevated expression of senescence markers SA-β-Gal, p16, p21 and SAHF marker H3K9me3. Consistent with a role of E6AP in PML degradation, lymphoma cells and premalignant B-lymphoid cells derived from *E*μ*-myc/E6AP*^*+/−*^ mice exhibit elevated levels of PML and PML-NBs compared with those cells derived from *E*μ*-myc* mice. Importantly, loss of one or both allele of PML significantly accelerates lymphoma development in *E*μ*-myc* mice model, supporting the contribution of PML elevation to the suppression of lymphomagenesis observed in *E*μ*-myc/E6AP*^*+/−*^ mice. Clinically, elevated E6AP expression is observed in human Burkitt lymphoma specimens and a number of B lymphoma cell lines and this elevated E6AP expression is associated with PML downregulation [43]. Thus, E6AP-mediated PML ubiquitination and degradation potentiates lymphomagenesis by inhibition of cell senescence and elevated E6AP expression likely contributes in part to PML downregulation in B cell lymphomas.

### Casein kinase 2 (CK2)-dependent PML ubiquitination

Besides CDK1/2, other kinases can also influence on PML ubiquitination and proteasomal degradation. One such kinase is CK2, a serine/threonine kinase with oncogenic potential [44]. CK2 phosphorylates PML at multiple sites. Among them, S517 (S565 in PML-I) is the prime phosphorylation residue and is within a PML degron. CK2 is known to be activated by p38MAPK during cell stress. In line with a role of CK2 in PML degradation, several stress conditions that activate CK2, such as osmotic shock and UV irradiation, potentiates PML proteasomal degradation. Inhibition of either p38MAPK or CK2 abrogates stress-induced PML degradation. Furthermore, PML ubiquitination is stimulated by a CK2-activating signal (i.e., osmotic shock) or by introducing a PML mutant that mimics the CK2 phosphorylation event [8]. These observations lead to the hypothesis that CK2 phosphorylation primes PML ubiquitination via an unidentified ubiquitin ligase. Interestingly, the CK2 phosphorylation residues are within an extended SIM and phosphorylation of these residues affects PML binding to sumoylated proteins [45,46]. The significance of CK2-dependent PML degradation in cancer biology is highlighted by the finding that PML phosphomimetic mutant acts as a super tumor suppressor due to its prolonged half-life. This mutant induces stronger senescence and apoptotic effects in cell-based assays and a more potent tumor suppressive function in a xenograft model, compared with wild type PML. In human cancers, aberrant activation of CK2 is found in a subset of non-small cell lung cancer (NSCLC) cell lines and patient specimens, which correlates with decreased PML protein levels [8,47]. Thus, the CK2-dependent PML degradation pathway is likely dysregulated in NSCLC to influence on tumor development and progression.

### ERK2-dependent PML proteasomal degradation

ERK2 is another PML kinase that stimulates PML proteasomal degradation. ERK2 is partially localized in PML-NBs and phosphorylates PML at S403 and S505 [48]. Similar to CDK1/2-induced PML phosphorylation, phosphorylation of PML by ERK2 facilitates the recruitment of Pin1, which promotes PML proteasomal degradation [49]. As protein degradation through proteasome is mainly mediated by ubiquitination, the ERK2- and Pin1-modified PML likely recruits an unidentified E3 ligase for its ubiquitination. ERK2 activity is stimulated by multiple growth factors, including EGF. Accordingly, EGF increases PML degradation through ERK2- and Pin1- dependent manner [48]. Given a frequent ERK2 activation in various types of human cancers, the ERK2/PML axis may contribute to PML degradation in certain cancers.

### Targeting PML ubiquitination pathway as anti-cancer strategies

In viewing of the pleiotropic functions of PML in tumor suppression and its frequent degradation in various types of cancers, targeting the PML ubiquitination/degradation pathway becomes an attractive approach for anti-cancer therapy. In theory, several approaches can be used for screening small molecular inhibitors that blocks PML ubiquitination pathways. The first one is to target the ubiquitin E3 ligases. Of note, a number of high throughput screening methods have been established for identifying inhibitors of E3 ligases [50]. For multi-subunit E3 ligases, the screen strategy can be designed for the disruption of interaction between subunits. The second approach is to screen small molecular inhibitors that disrupt the interaction of PML with its E3 ligases. Finally, since PML posttranslational modifications, such as phosphorylation and sumoylation, are often required for the subsequent ubiquitination events, targeting such posttranslational modification processes (e.g., by introducing a kinase inhibitor) can also prevent PML ubiquitination. In line with this notion, CK2 inhibitor emodin suppresses tumor growth in a xenograft model and this effect depends on the presence of a CK2 phosphorylation residue on PML [8]. The Cullin-family E3 ligase inhibitor MLN4924, which inhibits Cullin neddylation by targeting NEDD8 activating enzyme, elicits profound anti-tumor effects [51]. It would be intriguing to test whether MLN4924 attenuates prostate cancer progression induced by KLHL20-mediated PML degradation. Importantly, recent evidence has emerged that multiple pathways govern PML degradation in tumors and different types of cancers likely use distinct mechanisms to regulate PML stability. Thus, a full understanding of PML ubiquitination mechanisms in different cancer types and cellular conditions would aid in the design and selection of specific anti-cancer strategies for treating cancer patients displaying aberrant PML degradation.

## Conclusions

**Figure 1:**
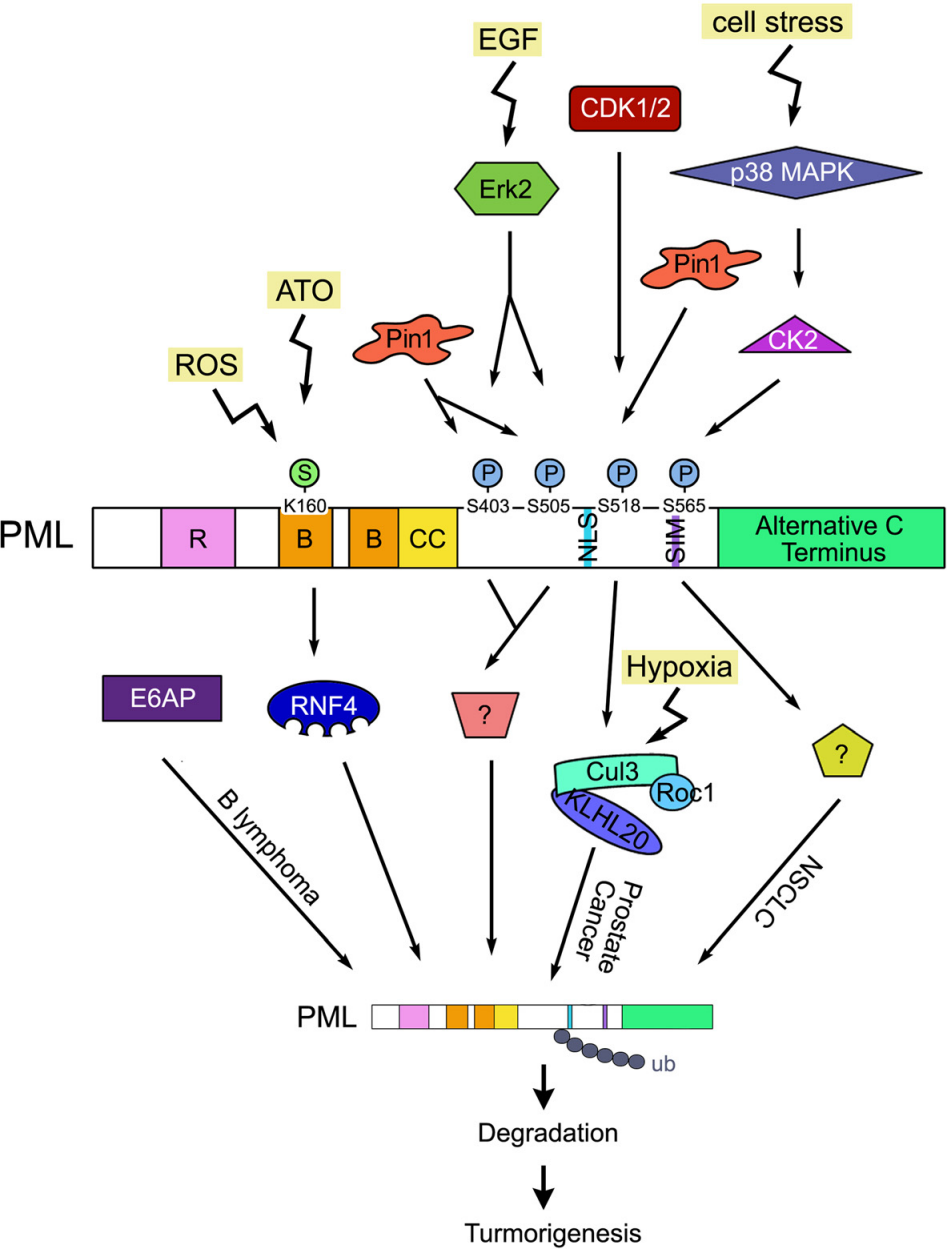
**Summary of the PML ubiquitination/degradation pathways.** These pathways are activated by extracellular or intracellular stimuli, primed by distinct PML posttranslational modifications, mediated by indicated E3 ligases, and perturbed in different types of human cancers. Unidentified E3 ligases are shown by question marks.

The ubiquitin-proteasome system plays a key role in maintaining cellular homeostasis and aberrant ubiquitin-dependent degradation of tumor suppressor proteins contributes to human tumorigenesis. The PML tumor suppressor protein is frequently downregulated in human cancers through a proteasome-dependent mechanism. Recent studies have revealed multiple pathways that lead to PML ubiquitination and degradation. These pathways involve distinct ubiquitin ligases and are often primed by different PML posttranslational modifications. Thus, it is conceivable that each PML ubiquitination pathway is stimulated under distinct cellular conditions, such as hypoxia, ROS, growth factors, and cell stress (Figure 1). Although aberrant regulation of some of these PML ubiquitination pathways has been observed in certain types of cancers, the prevalent PML degradation in cancers suggest the existence of additional ubiquitination pathways and/or regulatory mechanisms. Furthermore, the mechanism of PML deubiquitination, a process opposing ubiquitination, remains unexplored. It is important to further elucidate the regulatory mechanisms of PML ubiquitination in human cancers. As our understanding of this event progresses, more specific inhibitors targeting a particular pathway will be discovered to antagonize a particular subset of human cancers displaying aberrant PML degradation.

## Abbreviations

APL, acute promyelocytic leukemia; ATO, arsenic trioxide; CK2, casein kinase 2; IP3R, inositol 1,4,5-triphosphate receptor; PML, promyelocytic leukemia; PML-NBs, : PML-nuclear bodies; RBCC, Ring, B-box, and coiled-coil; SAHF, senescence-associated heterochromatin foci; SIMs, SUMO-interacting motifs; STUbLs, SUMO targeted ubiquitin ligases.

## Competing interests

The authors declare that they have no competing interests.

## Authors’ contributions

WCY and YRL were involved in drafting part of the manuscript and preparing figure. RHC collected information, designed the concept, and prepared the manuscript. All authors read and approved the final manuscript.
